# Effect of Aging on Change of Intention

**DOI:** 10.3389/fnhum.2019.00264

**Published:** 2019-07-31

**Authors:** Ariel Furstenberg, Callum D. Dewar, Haim Sompolinsky, Robert T. Knight, Leon Y. Deouell

**Affiliations:** ^1^Racah Institute of Physics, Faculty of Science, The Hebrew University of Jerusalem, Jerusalem, Israel; ^2^Helen Wills Neuroscience Institute, University of California, Berkeley, Berkeley, CA, United States; ^3^College of Medicine, University of Illinois at Chicago, Chicago, IL, United States; ^4^Edmond & Lily Safra Center for Brain Sciences, The Hebrew University of Jerusalem, Jerusalem, Israel; ^5^Department of Psychology, University of California, Berkeley, Berkeley, CA, United States; ^6^Psychology Department, The Hebrew University of Jerusalem, Jerusalem, Israel

**Keywords:** aging, intention, free-choice, decision making, masked-priming, EEG, LRP, EMG

## Abstract

Decision making often requires making arbitrary choices (“picking”) between alternatives that make no difference to the agent, that are equally desirable, or when the potential reward is unknown. Using event-related potentials we tested the effect of age on this common type of decision making. We compared two age groups: ages 18–25, and ages 41–67 on a masked-priming paradigm while recording EEG and EMG. Participants pressed a right or left button following either an instructive arrow cue or a neutral free-choice picking cue, both preceded by a masked arrow or neutral prime. The prime affected the behavior on the Instructed and the Free-choice picking conditions both in the younger and older groups. Moreover, electrophysiological “Change of Intention” (ChoI) was observed via lateralized readiness potential (LRP) in both age groups – the polarity of the LRP indicated first preparation to move the primed hand and then preparation to move the other hand. However, the older participants were more conservative in responding to the instructive cue, exhibiting a speed-accuracy trade-off, with slower response times, less errors in incongruent trials, and reduced probability of EMG activity in the non-responding hand. Additionally, “Change of Intention” was observed in both age groups in slow RT trials with a neutral prime as a result of an endogenous early intention to respond in a direction opposite the eventual instructing arrow cue. We conclude that the basic behavioral and electrophysiological signatures of implicit ChoI are common to a wide range of ages. However, older subjects, despite showing a similar dynamic decision trajectory as younger adults, are slower, more prudent and finalize the decision making process before letting the information affect the peripheral motor system. In contrast, the flow of information in younger subjects occurs in parallel to the decision process.

## Introduction

In order for decisions to play a significant role in human and animal behavior they have to be dynamic and contain the possibility for changes of choice, to enable continuous adjustment to the changing world (Cisek, 2007; Pezzulo and Cisek, 2016). Recent studies have documented the phenomenon of Change of Intention (ChoI) in controlled experimental conditions within a proximal decision process lasting seconds or less^1^ (e.g., Resulaj et al., 2009; Song and Nakayama, 2009; Kiani et al., 2014). This phenomenon poses a challenge to the simple prevalent model of decision making as a discrete irreversible event of threshold crossing of a noisy evidence accumulator of an internal signal (Bowman et al., 2006; Bogacz et al., 2007; Resulaj et al., 2009; Mattler and Palmer, 2012; Schurger et al., 2012; Fleming et al., 2018; Khalighinejad et al., 2018). Another challenge is raised by the observation of early biases in muscle activation, during the still ongoing process of decision making (Selen et al., 2012), which are sometimes incongruent with the final decision (Furstenberg et al., 2015).

The situation becomes more complex when a decision is required between arbitrary choices that make no difference to the agent, that are equally desirable, or when the potential reward is unknown (“picking”; Ullmann-Margalit and Morgenbesser, 1977). In such cases there is no a priori correct choice, raising the question about the nature of temporal dynamics that form intentions under these situations. In a previous study (Furstenberg et al., 2015) we showed, through behavioral and electrophysiological measurements performed on young adults (around 23 years old), that the decision process in a proximal *picking* selection task is often complex, continuous and dynamical, and may include also a ChoI pattern. Participants were required to press the left or right button, either according to an instructing left or right arrow cue or freely picking between left and right buttons following a free-choice cue. Prior to presenting the cue, in some trials a masked prime in the form of an arrow was presented. In that study three main phenomena stood out: (a) the subliminal arrow prime induced motor cortex activation [measured with EEG via lateralized readiness potential (LRP)], creating more activation in the motor cortex contralateral to the hand represented by the invisible prime arrow direction; this can be described as a positive compatibility effect (PCE; Neumann and Klotz, 1994; Dehaene et al., 1998; Eimer and Schlaghecken, 2003; Schlaghecken and Eimer, 2004; Kiesel et al., 2006); (b) Although the Free-choice condition was a picking scenario in which the alternatives make no difference to the participants, in many trials the participants chose the hand not cued by the prime. The LRP results were consistent with a ChoI scenario (previously shown in non-Free-choice cases, e.g., Dehaene et al., 1998; Gratton et al., 1988; Leuthold and Kopp, 1998; Jaśkowski et al., 2002). That is, the polarity of the LRP indicated first preparation to move the primed hand and then preparation to move the other hand, suggesting that the subjects overrode the intention induced by the prime [compare to Free-choice Go/No-Go results in Parkinson and Haggard (2014)]; and (c) Moreover, it was shown that in a portion of the incongruent trials the prime induced muscle activation (measured with EMG) of the hand incongruent to the hand that finally acted. The purpose of the present study was to investigate the influence of aging on these phenomena.

Aging causes a decline of many cognitive faculties (Hasher et al., 1999), including executive functions, inhibitory control, and motor functions (Dustman et al., 1996; Fujiyama et al., 2012; Levin et al., 2014), leading some to hypothesize that aging causes a general deficit in inhibition, as proposed in Hasher and Zacks (1988) seminal paper. For example, while masked primes induce an early PCE, with longer prime-cue inter-stimulus intervals (ISIs) they may also induce a negative compatibility effect (NCE) – a bias toward the direction incongruent with the prime, an RT delay and higher error rates for congruent responses, which is assumed to result from self-inhibition (e.g., Bowman et al., 2006) or from mask induced inhibition (e.g., Jaśkowski, 2007; Verleger, 2011). Studies have shown that while PCE is preserved in aging populations, older participants did not show a NCE, suggesting impaired or sluggish inhibition in older subjects [Seiss and Praamstra, 2004 (age range 58 ± 7); Schlaghecken and Maylor, 2005 (age range 71–83)]. However, Sumner et al. (2007) reported robust NCEs in a group of older adults (age range 56–75). Refining the claims, Schlaghecken et al. (2011) (age range 65–83) showed that low-level inhibitory processes (NCEs) existed in some instances, but were nevertheless delayed, more so than expected from general age-related slowing of RT [similar conclusion in Seiss and Praamstra (2006) (age range 53.6 ± 4.9) reinterpreting their earlier results]. In addition, there was large inter-individual variability. Finally, a recent meta-analysis [Rey-Mermet and Gade (2017); see also discussion in Kramer et al. (1994)] called into question the hypothesis of a general age-related weakening of inhibitory processes, showing that in many tasks, older adults do not exhibit such impairments. In any case, the shift from PCE to NCE is a clear manifestation of ChoI, which is the focus of the current study.

The goal of the present study was to investigate the effect of aging on the process of forming intentions in a *picking* situation and on the pattern of responses to external *instructing* cues, using the masked prime paradigm of our previous study. Specifically, we aimed at illuminating two aspects: (1) Do the older participants express “change of intention” to the same extent as the younger group? (2) Do the older participants manifest the same continuous information flow, expressed in early incongruent muscle activation, as was shown in the younger group?

## Materials and Methods

### Participants

Three groups participated in the experiment. We first studied one younger group (“Young1”; *N* = 15, 6 females, mean age = 20.6, SE = 0.5; age range: 18–24) and one older group (“Older1”; *N* = 12, 9 females, mean age = 55.1, SE = 2.4; age range: 41–67). As will be elaborated below, the results showed reduced masked priming effects in the older group. We surmised that this was due to the very short duration of prime presentation used. Since the presence of masked priming was a sine qua non for answering the research question, we recruited a second group of older participants (“Older2”; *N* = 14, 8 females, mean age = 56.8, SE = 1.8; age range: 42–65) to whom the prime was presented for a slightly longer duration (see section “Procedure” below). In order to obtain a more comprehensive picture we also included the results of another young group that we previously reported in Furstenberg et al. (2015). This group of young participants (“Young2”; *N* = 14, 8 women, mean age = 23.8, SE = 0.7) had performed the same task (for more details please consult the original article).

All participants reported normal or corrected-to-normal vision and no history of neurological disorders. Participants received monetary compensation or course credits for their participation in the study. Written consent was obtained after the general experimental procedures were explained. The study was approved by the Ethics Committee of the University of California, Berkeley.

### Stimuli

**FIGURE 1:**
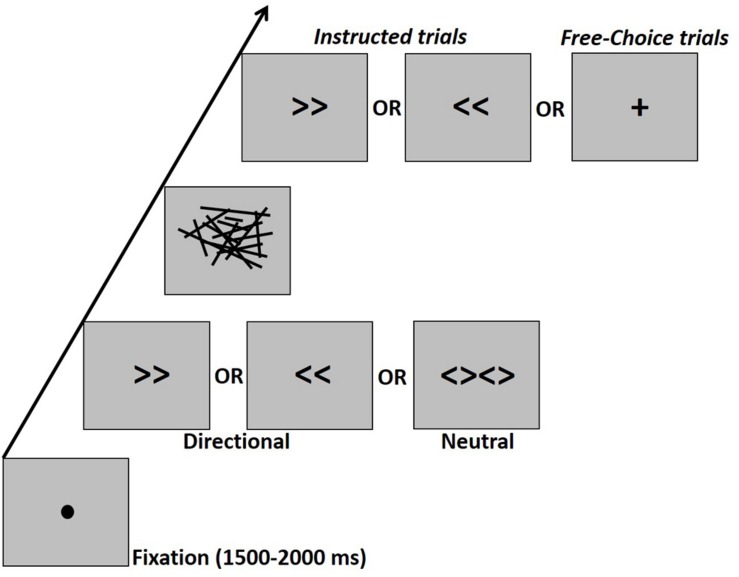
Schematic experimental design. Participants had to press the right or left button according to an instructive arrow cue or according to their “free-choice.” The cue was preceded by a masked prime. The prime-mask-cue sequence was presented, respectively for durations of 17–33–67 ms in the Young1 and Older1 groups, for 27–40–67 ms in the Older2 group, and for 20–40–70 ms in the Young2 group. There were no gaps between prime, mask, and target, so that the prime-target ISI was fully occupied by the mask.

Prime stimuli included the symbols “>>” (right directional prime, visual angle: 2.8° × 1.24°), “<<” (left directional prime, 2.8° × 1.24°) or “<><>” (neutral prime, visual angle: 5.68° × 1.24°). The mask was constructed of lines of various lengths and orientation scattered within a virtual rectangle (visual angle: 13.4° × 7.6°). There were eight such different masks which were randomly applied. The cue was a right or left double arrow identical to the directional prime arrows or a “+” sign (visual angle: 2.23° × 2.23°) which indicated a free-choice trial (Figure 1).

### Procedure

The experiment took place in a dimly lit, sound attenuated room. Participants were comfortably seated in front of a CRT monitor (ViewSonic P220f; groups Young1 and Older1: 60 Hz refresh rate; Young2: 100 Hz refresh rate; Older2: 75 Hz refresh rate), with a viewing distance of 105 cm. All stimuli were presented at the center of the screen in black color on a gray background. Responses were provided through a standard computer keyboard placed on the participant’s lap. Experimental procedure was controlled by a PC running E-Prime software (Psychology Software Tools, Sharpsburg, PA; version 2.0). Trials started with a fixation point (filled black circle, visual angle: 0.38°) for 1500–2000 ms (uniform distribution with 100 ms steps), followed by the presentation of a prime for 17 ms (one screen refresh at 60 Hz) in groups Young1 and Older1, 27 ms (2 screen refreshes at 75 Hz) in the Older2 group, and 20ms in the Young2 group (2 screen refreshes at 100 Hz). The prime was followed immediately by a backward mask for 33 ms (2 screen refreshes at 60 Hz) in the first two groups and 40 ms (3 screen refreshes at 75 Hz, or four refreshes at 100 Hz) for Older2 and Young2, and finally the cue appeared for 67 ms for all groups except Young2 in which it appeared for 70 ms (Figure 1). There were no gaps between prime, mask, and target, so that the prime-target ISI was fully occupied by the mask. The task was to press a button as fast and accurately as possible upon seeing the cue with the left or right index finger (on the “z” and “/” QWERTY keyboard buttons, respectively), according to the information given by the cue. In the “Instructed” condition participants had to press with the hand that corresponds to the direction of the cue (i.e., left hand for left pointing arrow and vice-versa). In the Free-choice condition participants had to press with either the left or right finger as they choose. Note that Instructed and Free-choice trials were randomly mixed and until the onset of the cue, participants did not know whether the trial is Instructed or Free-choice.

To motivate fast responses, the participants had a response window of 867 ms from cue onset. In trials in which responses exceeded these limits, a “too slow” message was presented, and the reaction time (RT) of these trials was discarded from later analyses. An incorrect response to an Instructed arrow cue resulted in an error message “Wrong” and a somewhat unpleasant tone.

The experiment consisted of 8 blocks, each composed of 35 Instructed and 35 Free-choice trials with arrow primes, plus 35 Instructed and 35 Free-choice trials with neutral primes (all together 140 trials per block). In Instructed trials with arrow primes, the cue had equal probability to be left or right, and orthogonally, the prime had equal probability to be left or right. Thus, there were equal chances for the prime to be congruent or incongruent with the cue. In Free-choice trials with arrow primes the prime had also equal probability to point left or right. Two practice blocks preceded the experiment, a short one with a mixture of 16 Instructed and 16 Free-choice trials in which the experimenter accompanied the participant and commented on performance if needed, and a second extended one with a mixture of 60 Instructed and 60 Free-choice trials all with arrow primes, where participants were on their own in the experimental booth. The experimental blocks thus started after the participants became confident with the task and stimuli. The participants were encouraged to rest their eyes for a few minutes between blocks.

### Behavioral Data

#### Objective Evaluation of Awareness

Following the main experimental blocks, awareness of the primes was evaluated in an objective 2-interval 2-alternative-forced-choice (2AFC) testing block. Each trial consisted of two different masking screens (each randomly chosen as above) appearing one after the other (each at an equivalent duration as in the testing blocks) with an ISI of 1.5 s. One of these masking screens (randomly chosen in each trial) was preceded by an arrow prime (identical to the one used in the main experiment and with an equivalent duration), while the other masking screen was preceded by a filler stimulus consisting of two slanted lines (“//”; visual angle: 2.76° × 0.6°) with same duration. Participants were asked successively (1) whether a rapid arrow prime appeared before the first or before the second mask in a 2-alternative forced choice manner (2AFC; detection), and (2) whether the arrow prime pointed left or right (2AFC; discrimination). The first question (detection) was replied by pressing with the left hand on the “1” or “2” top keyboard keys, and the second question (discrimination) was replied by pressing with the right hand on the keyboard left or right arrow keys. Emphasis was given to accuracy and not to speed. The block consisted of 50 trials. Each trial began after the participant replied to the two successive questions of the previous trial. Twice during the block (after 20 and after 40 trials) participants were presented with a feedback screen with the number of successful detection and discrimination hits. The purpose of this feedback was to keep the participants motivated.

#### Behavioral Analysis

A 2-way analysis of variance was done with factors Congruency (congruent, incongruent, neutral; within-subject) and Group (Young1, Young2, Older1, Older2; between-subjects) for Instructed RT and error rates and for Free-choice RT. Where the sphericity assumption was violated, we report Greenhouse-Geisser corrected *p*-value and uncorrected degrees of freedom. In the Instructed trials the congruent trials were defined as those with similar prime and cue, incongruent trials as those with opposing prime and cue and neutral trials as those with a neutral prime. In the Free-choice trials no explicit directional cue was given, thus we define congruency according to the match between the direction of the prime and the chosen response. Bias of choices toward the prime direction was measured by the percentage of congruent choices out of all choices. As prime direction was equally distributed between left and right, a pre-existing response bias for one direction would not be reflected in a congruency effect (e.g., if in the extreme case the participant pressed only with one hand, there would be no congruency bias). By definition, there were no errors in the Free-choice condition.

For the awareness evaluation block, we calculated for each participant the percent of correct answers in the detection task. Participants were categorized as “aware of the prime” if their score was significantly above chance level of 50%, defined as being in the top 5% of the sampling distribution of proportion that is centered around 50%. The critical above-chance proportion *P*_c_ was calculated based on the sampling distribution of proportion:

$$p_{c}=p_{0}+Z_{1-\alpha }\times \sqrt{\frac{p_{0}\,\left(1-p_{0}\right)}{n}}$$

with *p*_0_ = 0.5; *n* = number of trials, and α = 0.05, resulting in a cutoff of *p*_c_ = 0.617. Participants which were categorized in the detection task as “aware of the prime” were excluded from further analysis.

### Electrophysiological Data

#### EEG Recording

EEG was recorded continuously with pre-amplified sintered Ag/AgCl electrodes from 64 scalp locations according to the extended 10–20 system^2^, as well as from the tip of the nose, using a BioSemi Active 2 system (Biosemi, Netherlands). Blinks and eye movements were monitored using two EOG electrodes located at the outer canthi of the right and left eyes for horizontal movements, and an electrode below the center of the right eye together with the Fp2 electrode for vertical movements. The EEG was sampled at 1024 Hz with an online low-pass anti-aliasing filter (cutoff: 205 Hz) and stored for off-line analysis. Analysis was conducted using Brain Vision Analyzer 2 (Brain Products, Germany), and MATLAB R2016b (The MathWorks Inc., Natick, MA). The EEG data was digitally referenced to the nose and filtered with a bandpass of 0.5–15 Hz (zero-phase shift, 24 dB/octave Butterworth filter) for the ERP analysis. Blink artifacts were removed using the independent component analysis (ICA) method (Jung et al., 2000; as implemented in Analyzer 2). Segments contaminated by other artifacts were discarded (rejection criteria: >100 μV absolute difference between samples within intervals of 100 ms; absolute amplitude beyond the ±100 μV range). Participants were asked to minimize movements and eye blinks during the task.

#### ERP Analysis

The continuous EEG signal was segmented into single trial epochs, separately for the experimental conditions of Prime Direction (Right/Left/Neutral) × Cue (Right arrow/Left arrow/Free-choice) × Response Direction (Right/Left). As a convention, we refer to specific conditions using a three letter code composed of the first letters of the prime, cue and response identities, with + as the identifier of the Free-choice condition (e.g., RRR for right prime, right cue, right response, and L + R for left prime, Free-choice cue, right response). In instructed trials, only correct responses were analyzed, thus response direction is identical to cue direction in these trials. We used the LRP event-related potential in order to identify preparation of left or right hand movement. The LRP was derived by subtracting the pre-response potential recorded from a central, parasagittal scalp electrode, roughly located over the right motor cortex (C4) from the potential recorded at a homologous site over the contralateral scalp (C3). Within this framework, since the potential over the motor cortex contralateral to the action hand becomes more negative, activation of the left motor cortex in preparation of right hand movement results in a negative shift of the LRP, whereas activation of the right motor cortex in preparation to move the left hand results in a positive shift of the LRP (de Jong et al., 1988; Coles, 1989).

One of our main goals was to examine the effect of the prime and the cue on the formation of intentions in the Instructed and in the Free-choice conditions within the different age groups. Since RTs vary from trial to trial, prime and cue-dependent information might not be apparent in response-locked averages due to temporal smearing. Thus, we analyzed the LRP locked to the prime onset, emphasizing the stimulus-dependent information. For this analysis, segments extended from 200 ms before till 1000 ms after prime onset. Potentials were calculated relative to a (−200) to (−50) ms pre-stimulus baseline period. We obtained LRPs for each group comparing congruent and incongruent trials within Instructed and Free-choice conditions. We pooled the LRP across trials with right and left hand responses by reversing the direction of subtraction for left hand:

$${\mathrm{LRP}}_{\mathrm{across}\,\mathrm{hands}}=\frac{\sum_{\mathrm{right}\,\mathrm{response}\,\mathrm{trials}}\left(C_{3}-C_{4}\right)+\sum_{\mathrm{left}\,\mathrm{response}\,\mathrm{trials}}\left(C_{4}-C_{3}\right)}{\mathrm{Number}\,\mathrm{of}\,\mathrm{trials}}$$

Thus, a negative deflection of the LRP_across_ _hands_ represents preparation commensurate with the final button press (whether left or right), whereas a positive deflection represents preparation in the opposite direction. This equation yields the highest SNR for the LRP, as it weighs properly left hand and right hand responses, according to their frequency. However, in cases where the number of left hand and right hand response trials were not equal (as in the case of the free choice conditions) this method of calculating the LRP may be contaminated by permanent asymmetry between the hemispheres which is not contingent on the acting hand (Coles, 1989; Oostenveld et al., 2003). Hence, to ensure that the effects we find are not due to such a permanent asymmetry, we also calculated the LRPs by first averaging separately right hand and left hand response trials, and then averaging these averages (i.e., an unweighted average):

Unweighted LRPacross⁢hands=12×(∑right⁢response⁢trials(C3-C4)Number⁢of⁢right⁢trials+∑left⁢response⁢trials(C4-C3)Number⁢of⁢left⁢trials)

We used a cluster based permutation test to test for significant differences between conditions without prior assumptions about the timing of the effect. Specifically, following Maris and Oostenveld (2007), we first performed point-by-point *t*-tests comparing two conditions. Then, for each cluster of consecutive time points in which the *t*-test yielded a significant difference (*p* < 0.05, uncorrected), we calculated a *t*-sum statistic, defined as the sum of all *t*-values of the cluster. This statistic is affected by both the strength and temporal extent of a cluster. To estimate the probability of finding a cluster with a given *t*-sum value in case that the null hypothesis of no difference between conditions is true, we performed a permutation analysis. This analysis included 10,000 iterations of the *t*-cluster analysis. For each iteration, we randomly permuted the condition labels within participant and obtained the maximal *t*-cluster statistic. Finally, a cluster of consecutive significant *t*-tests in the real data was considered to be significant only if its *t*-sum statistic exceeded the 95% cutoff of this null distribution of max *t*-sum values. We used this approach to compare the incongruent condition to the congruent condition. The main difference found in our previous study between the congruent and incongruent LRPs was that the incongruent trials expressed a “change of intention” signature (Furstenberg et al., 2015). With this in mind we compared in this study the different age groups.

Another ERP analysis for all age groups was dividing the neutral Instructed trials (NRR, NLL) into fast and slow trials (median split) and plotting the LRP for the fast vs. the slow trials, pooling left and right responses as explained above. This analysis was motivated by the hypothesis that in the neutral cases there is no prime to create an initial movement preparation (“early intention”), however, in various trials there might be a different inner endogenous inclination to move one way or the other (“inner bias”) which is then taken over by the instructing arrow cue which the participant complies with. Therefore, we hypothesized that the difference between slow and fast trials is that slow trials are caused, at least partially, by an early endogenous intention to move the hand in one direction which turns out to be in the direction opposite to the instructing arrow cue, thus creating a ChoI signature. If this were the case, we expected the difference between the neutral fast and slow LRPs to resemble the difference between congruent and incongruent LRPs. The difference between the LRP signals was measured again with a permutation test as described above in the different age groups.

#### EMG Recording and Analysis

The EMG was recorded continuously and synchronously with the EEG (same recording system and parameters) by placing two Ag/AgCl electrodes, proximal and distal, about 5cm apart, over the flexor digitorum superficialis muscle. For each hand, the muscle activation signal was calculated as the difference between the proximal and distal electrodes. Offline, the data was digitally filtered with a high-pass filter with a cutoff of 15 Hz (zero-phase shift, 48 dB/octave Butterworth filter) and segmented according to the experimental conditions (see “EEG recording” and “ERP analysis”). Muscle activity was extracted using a moving window (15 sample-points length) root-mean-square (RMS) over the EMG signal. Muscle activation was defined as RMS exceeding 4 standard deviations from the 150 ms baseline interval prior to prime onset. The onset of the EMG response was defined as the first sample point exceeding this threshold. In order to evaluate EMG activity without overt action, onset was calculated for left and right hand regardless of the hand executing the final button press.

Following earlier studies that compared EMG activity between incongruently primed hand and correct hand (e.g., Coles et al., 1985; Smid et al., 1990; Burle et al., 2008; Roger et al., 2014; Furstenberg et al., 2015), we examined ChoI in the EMG activity by testing whether an incongruent prime increased the probability for an EMG response in the non-responding hand relative to a congruent prime, in the different age groups. Since percentages do not distribute normally, we used within each group a permutation based test, in which the true difference between the average percentages of trials in the two conditions is compared against a permutation null distribution. This null distribution was constructed by performing 10,000 permutations, in each of which the labels of the conditions were randomly shuffled for each participant, and the resulting surrogate average difference between the percentages was registered. Another permutation test was done to determine the significance of a 2-way analysis of variance (based on Anderson and Braak, 2003; 10,000 permutations) with factors Congruency (congruent, incongruent; within-subject) and Group (young (pooled), older (pooled); between-subjects) for the probability for an EMG response in the non-responding hand.

EMG ChoI was also analyzed for the neutral Instructed fast and slow trials with the same logic as in the “ERP Analysis” section, comparing the younger and older groups. Thus, we tested in the different age groups the hypothesis that the neutral Instructed slow trials had increased probability for an EMG response in the non-responding hand relative to neutral Instructed fast trials. This was done by implementing the permutation test to test the significance of a 2-way analysis of variance (10,000 permutations) with factors RT (fast, slow; within-subject) and Group (young (pooled), older (pooled); between-subjects) for the probability for an EMG response in the non-responding hand.

## Results

### Objective Awareness Test

In the results of the main task described below we considered only participants who were unaware of the masked prime based on the objective awareness test.

*Young1:* Three out of 15 participants had higher than chance-level performance in the objective evaluation of awareness task (2AFC detection of the masked prime arrow), and were thus excluded from the analysis. The performance of each of the remaining 12 participants (3 females, mean age = 20.7, SE = 0.6) did not differ from chance level in the detection test. The mean of correct responses in the objective detection test in this group of participants was 47.7%, which was not significantly different from chance (*t*_(11)_ = 1.58, *p* = 0.07). In the left–right discrimination task, which was not part of our selection process, these participants scored an average of 50.5%, SE = 1.0, again suggesting that they did not see the primes in any meaningful way.

All the participants of the Young2 group included in the analysis passed the objective awareness test, as described in Furstenberg et al. (2015).

*Older1:* One out of 12 participants had higher than chance-level performance in the objective evaluation of awareness task (2AFC detection of the masked prime arrow), and was thus excluded from the analysis. The performance of each of the remaining 11 participants (9 females, mean age = 54.6, SE = 2.5) did not differ from chance level in the detection test. The mean of correct responses in the objective detection test in this group of participants was 51.5%, which was not significantly different from chance (*t*_(10)_ = 0.8, *p* = 0.22). In the discrimination task, which was not part of our selection process, these participants scored an average of 54%, SE = 1.1.

*Older2:* One out of 14 participants had higher than chance-level performance in the objective evaluation of awareness task (2AFC detection of the masked prime arrow), and was thus excluded from the analysis. The performance of each of the remaining 13 participants (8 females, mean age = 56.8, SE = 1.9) did not differ from chance level in the detection test. The mean of correct responses in the objective detection test in this group of participants was 47.4%, which was not significantly different from chance (*t*_(12)_ = 1.32, *p* = 0.11). In the discrimination task, which was not part of our selection process, these participants scored an average of 53.5%, SE = 1.2.

### Behavior

As a reminder, participants performed two types of trials (Figure 1; section “Procedure”). Instructed trials consisted of a masked prime (left or right arrow, or neutral stimuli consisting of two diamond shapes) followed by a left or right arrow cue. The task was to press the right or left button with the respective hand quickly and accurately according to the instructing cue. Free-choice trials consisted of a masked prime (as above), followed by a Free-choice cue (plus sign), and the task was to press the left or right button quickly according to the participant’s free-choice at the moment of the cue. In all trial types, the participants were not informed of the existence of the prime.

We ran a 2-way ANOVA with factors Congruency (congruent, incongruent, neutral; within-subject) and Group (Young1, Young2, Older1, Older2; between-subjects) separately for the Instructed RT, Free-choice RT, and Instructed error rates. The results showed a significant main effect of Congruency for all three measures (Instructed RT: *F*(2,92) = 164.7, *p* < 0.001, partial η^2^ = 0.78; Free-choice RT: *F*(2,92) = 74.6, *p* < 0.001, partial η^2^ = 0.62; Instructed error rate: *F*(2,92) = 27.8, *p* < 0.001, partial η^2^ = 0.38), expressing the fact that the congruent response is mainly faster than the incongruent response with the neutral in between, and that there are more errors in the incongruent trials compared to the congruent trials with the neutral in between. The results also showed a significant main effect of Group for the three measures (Instructed RT: *F*(3,46) = 28.1, *p* < 0.001, partial η^2^ = 0.65; Free-choice RT: *F*(3,46) = 15.1, *p* < 0.001, partial η^2^ = 0.5; Instructed error rate: *F*(3,46) = 6.9, *p* = 0.001, partial η^2^ = 0.31), expressing the fact that the younger adults were much faster in their responses than the older adults, however, they had higher error rates in the incongruent trials than the older adults. These main effects were qualified, however, by significant interactions between Group and Congruency (Instructed RT: *F*(6,92) = 9.0, *p* < 0.001, partial η^2^ = 0.37; Free-choice RT: *F*(6,92) = 12.3, *p* < 0.001, partial η^2^ = 0.44; Instructed error rate: *F*(6,92) = 3.4, *p* = 0.004, partial η^2^ = 0.18). To interpret the interaction of Group and Congruency, we performed 1-way ANOVA with the Congruency factor (congruent, incongruent, neutral) for each group, followed by planned comparisons based on the comparisons previously done in Furstenberg et al. (2015).

**FIGURE 2:**
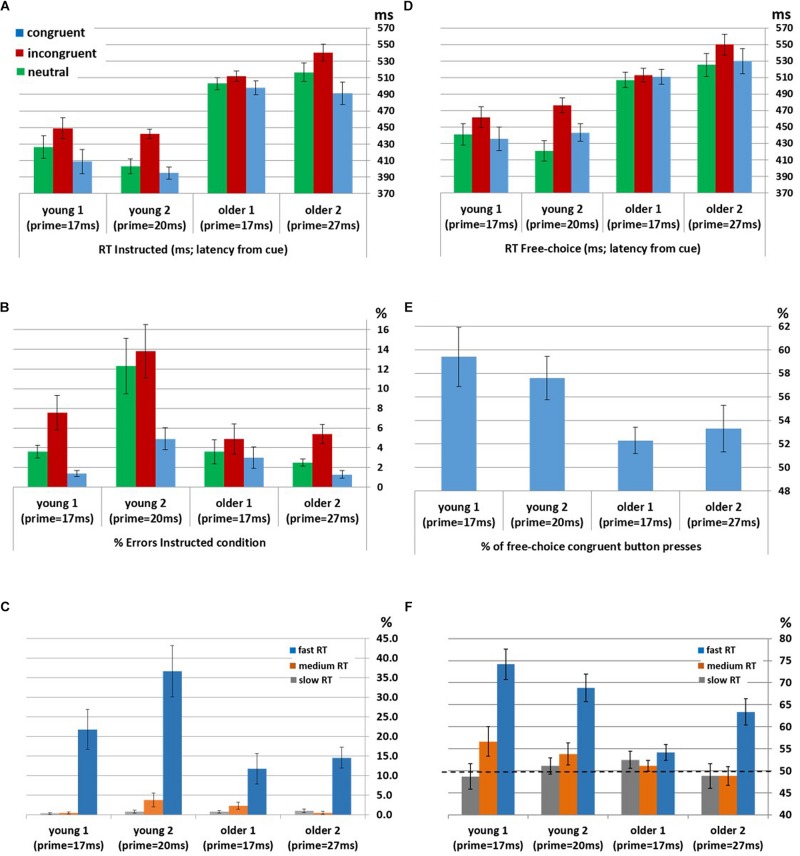
Behavioral priming effect in participants unaware of the prime within each group (Young1, Young2, Older1 and Older2). **(A)** Mean RT in Instructed trials. **(B)** Error rates in Instructed trials. **(C)** Error rates in Instructed incongruent trials by RT bins (fast, medium, and slow). **(D)** Mean RT in Free-Choice trials. **(E)** The average percentage of pressing the button congruent with the prime in Free-Choice trials, showing the bias toward the prime. **(F)** The average percentage of pressing the button congruent with the prime in Free-Choice trials by RT bins (fast, medium, and slow). All error bars denote the standard deviation of the error variance (standard error). RT is measured here from the onset of the cue.

Instructed RTs: Significant congruency effects were found for the Young1 group [*F*(2,22) = 54.0, *p* < 0.001, partial η^2^ = 0.83], Young2 [*F*(2,26) = 81.1, *p* < 0.001, partial η^2^ = 0.86], Older1 [*F*(2,20) = 11.5, *p* = 0.003, partial η^2^ = 0.53, Greenhouse-Geisser epsilon = 0.66], and Older 2 [*F*(2,24) = 44.8, *p* < 0.001, partial η^2^ = 0.79]. Follow up contrasts showed that, like we previously found in the Young2 group, these effects stem from a PCE in the Instructed condition (Figure 2A and Table 1): RTs were slower for incongruent compared to congruent trials (Young1: *t*_(11)_ = 8.24, *p* < 0.001, Cohen’s *d* = 2.38; Older1: *t*_(10)_ = 3.64, *p* = 0.002, Cohen’s *d* = 1.1; Older2: *t*_(12)_ = 7.05, *p* < 0.001, Cohen’s *d* = 1.96; all *p*-values are 1-tailed as we had a directional hypothesis). Neutral trials were significantly slower than congruent trials (Young1: *t*_(11)_ = 5.99, *p* < 0.001, Cohen’s *d* = 1.73; Older1: *t*_(10)_ = 1.9, *p* = 0.043, Cohen’s *d* = 0.57; Older2: *t*_(12)_ = 5.59, *p* < 0.001, Cohen’s *d* = 1.55), and significantly faster than incongruent trials (Young1: *t*_(11)_ = 6.37, *p* < 0.001, Cohen’s *d* = 1.84; Older1: *t*_(10)_ = 4.3, *p* < 0.001, Cohen’s *d* = 1.3; Older 2: *t*_(12)_ = 6.85, *p* < 0.001, Cohen’s *d* = 1.9). Thus, while the 2-way ANOVA revealed an interaction, likely stemming from the weaker effects in the Older1 group (Figure 2A, and see below), the PCE was evident in all groups.

**TABLE 1:** Mean reaction times (ms) and error rates (%) for congruent, incongruent, and neutral trials in Instructed and Free-Choice conditions for Young1, Older1, and Older2 groups.

	**Instructed**						**Free-choice**		
	**Mean RT (ms) (SE)**			**Error rates (%) (SE)**			**Mean RT (ms) (SE)**		
	**Congruent**	**Incongruent**	**Neutral**	**Congruent**	**Incongruent**	**Neutral**	**Congruent**	**Incongruent**	**Neutral**
Young1	409 (14.5)	449 (12.4)	426 (13.7)	1.4 (0.31)	7.6 (1.76)	3.6 (0.63)	435 (14.3)	462 (12.7)	441 (12.8)
Older1	498 (8.2)	513 (6.4)	503 (7.4)	3 (1.08)	4.9 (1.52)	3.6 (1.23)	511 (9)	513 (8.3)	507 (9.3)
Older2	491 (13.5)	540 (10.2)	517 (11.3)	1.3 (0.38)	5.4 (0.97)	2.5 (0.37)	530 (15.1)	550 (12.8)	525 (13.9)

Instructed Error rates: Significant congruency effects were found for the Young1 group [*F*(2,22) = 11.4, *p* = 0.005, partial η^2^ = 0.51], Young2 [*F*(2,26) = 12.1, *p* = 0.001, partial η^2^ = 0.48], Older 2 [*F*(2,24) = 11.0, *p* = 0.005, partial η^2^ = 0.48], but not in Older1 [*F*(2,20) = 1.9, *p* = 0.2, partial η^2^ = 0.16]. Follow up contrasts showed that for the Young1 group, the error rates were significantly higher in the incongruent compared to congruent condition (1-tailed *t*-test; *t*_(11)_ = 3.49, *p* = 0.002, Cohen’s *d* = 1.01; Table 1 and Figure 2B), as was the case in our previous report of Young2 group. Error rates in the neutral condition in the Young1 group were also intermediate: significantly higher than the congruent condition and lower than the incongruent conditions (*t*_(11)_ = 3.58, *p* = 0.002, Cohen’s *d* = 1.03 and *t*_(11)_ = 3.1, *p* = 0.005, Cohen’s *d* = 0.89, respectively). For the Older1 group with the same prime and mask durations these trends were not significant (1-tailed *t*-test; congruent vs. incongruent: *t*_(10)_ = 1.42, *p* = 0.1; congruent vs. neutral: *t*_(10)_ = 1.28, *p* = 0.12; neutral vs. incongruent: *t*_(10)_ = 1.3, *p* = 0.11). In contrast, for Older2 group, error rates were significantly higher in the incongruent compared to congruent condition (*t*_(12)_ = 3.46, *p* = 0.002, Cohen’s *d* = 0.96; Table 1 and Figure 2B), and in the neutral condition they were intermediate and differed significantly from the congruent and the incongruent conditions (*t*_(12)_ = 3.46, *p* = 0.002, Cohen’s *d* = 0.96 and *t*_(12)_ = 3.1, *p* = 0.005, Cohen’s *d* = 0.86, respectively). We further calculated the error rates for three separate bins of trials by trisecting the range of RTs representing the fastest, medium and slowest RTs. As would be expected from a speed-accuracy tradeoff, most of the errors were found in trials with faster RTs for all groups (Figure 2C).

Free-choice: Significant RT congruency effects were found for the Young1 group [*F*(2,22) = 34.9, *p* < 0.001, partial η^2^ = 0.76], Young2 [*F*(2,26) = 40.6, *p* < 0.001, partial η^2^ = 0.76], Older2 [*F*(2,24) = 21.6, *p* < 0.001, partial η^2^ = 0.64], but not in Older1 [*F*(2,20) = 2.2, *p* = 0.15, partial η^2^ = 0.18]. In the Young1 group, RTs were significantly slower when freely picking a response incongruent with the prime than when picking a response congruent with the prime (1-tailed *t*-test; *t*_(11)_ = 6.61, *p* < 0.001, Cohen’s *d* = 1.91; Table 1 and Figure 2D), and the RTs following neutral primes were intermediate between those following congruent and incongruent primes (incongruent slower than the neutral: 1-tailed *t*-test, *t*_(11)_ = 7.14, *p* < 0.001, Cohen’s *d* = 2.06; neutral slower than congruent,1-tailed *t*-test; *t*_(11)_ = 1.86, *p* = 0.045, n.s.^3^ Cohen’s *d* = 0.54). In the Older1 group, while the trends were similar, none of these effects were significant (congruent vs. incongruent RT: *t*_(10)_ = 0.63, *p* = 0.27; congruent vs. neutral RT: *t*_(10)_ = 1.35, *p* = 0.1; incongruent vs. neutral RT: *t*_(10)_ = 1.78, *p* = 0.053). However, RTs were significantly slower for the Older2 group when freely picking a response incongruent with the prime than when picking a response congruent with the prime (1-tailed *t*-test; *t*_(12)_ = 4.6, *p* < 0.001, Cohen’s *d* = 1.28; Table 1 and Figure 2D). The incongruent trials were significantly slower than the neutral trials as well (1-tailed *t*-test; *t*_(12)_ = 5.78, *p* < 0.001, Cohen’s *d* = 1.6). The RT in the neutral condition tended to be faster than the congruent condition (*t*_(12)_ = 1.5, *p* = 0.08).

Choice bias: We tested the tendency to select the hand cued by the prime in the free choice condition. One-way between-groups ANOVA of Free-choice response bias showed a significant difference between groups [*F*(3,49) = 3.4, *p* = 0.025]. Although nominally the older groups showed lower bias then the younger groups, *post hoc* pairwise comparisons using the Tukey HSD test did not show significant effects between individual groups. Tested individually against no-bias (50%), response direction was significantly biased toward the prime direction in the young groups (1-tailed *t*-test; Young1: *M* = 59.4%, SE = 2.51, *t*_(11)_ = 3.74, *p* = 0.002; Young2: *M* = 57.6%, SE = 1.83, *t*_(13)_ = 4.15, *p* < 0.001, Figure 2E). In the older groups, response direction was biased toward the prime direction but the effect was not significant (1-tailed *t*-test; Older1:*M* = 52.3%, SE = 1.13, *t*_(10)_ = 2.04, *p* = 0.07; Older2: *M* = 53.3%, SE = 2, *t*_(12)_ = 1.6, *p* = 0.13). Previous observations showed that weak prime effects are diminished when responses are delayed (Kinoshita and Hunt, 2008; Avneon and Lamy, 2018) as indeed they were in the older compared to the younger groups. Therefore, in a *post hoc* analysis, we examined the congruency effect separately within the abovementioned RT bins (fast, medium and slow). We found that the percent of congruent responses in the Free-choice condition was significantly above chance for all groups within the fastest RT bin (Figure 2F and Table 2; RT distribution and marked trisecting points in Supplementary Figure S1).

**TABLE 2:** The average percentage of pressing the button congruent with the prime in Free-choice trials by RT bins (fast, medium, and slow) within the tested groups (Young1, Young2, Older1, and Older2) and summary of 1-tailed *t*-test comparing results to 50% chance level.

	**Fastest RT**	**Medium RT**	**Slowest RT**
Young1	74.16 (3.48);	56.63 (3.35);	48.7 (2.89);
	*t*_(11)_ = 6.87, *p* < 0.001	*t*_(11)_ = 1.97, *p* < 0.05	*t*_(11)_ = 0.45, *p* = 0.33
Young2	68.8 (3.12);	53.82 (2.5);	51.11 (1.86);
	*t*_(13)_ = 6.06, *p* < 0.001	*t*_(13)_ = 1.52, *p* = 0.08	*t*_(13)_ = 0.57, *p* = 0.28
Older1	54.18 (1.8);	51.11 (1.22);	52.5 (1.91);
	*t*_(10)_ = 2.29, *p* < 0.05	*t*_(10)_ = 0.89, *p* = 0.19	*t*_(10)_ = 1.37, *p* = 0.11
Older2	63.4 (3.01);	48.83 (2.11);	48.84 (2.76);
	*t*_(12)_ = 4.46, *p* < 0.001	*t*_(12)_ = 0.56, *p* = 0.3	*t*_(12)_ = 0.39, *p* = 0.34

*The standard error is in parenthesis.*

Taken together, the results show that the Older1 group has weaker effects than the other groups. As an additional confirmation, we compared the congruency effects of each of the older groups to the pooled young groups. Older2 group did not differ from the pooled young group in any of the measures: Instructed RT (Older2: *M* = 49.2 ms, SE = 6.9; young: *M* = 44 ms, SE = 3.3; *t*_(37)_ = 0.77, *p* = 0.45), Free-choice RT (Older2: *M* = 19.9 ms, SE = 4.3; young: *M* = 29.9 ms, SE = 4.0; *t*_(37)_ = 1.55, *p* = 0.13) and Instructed error rate (Older2: *M* = 4.1%, SE = 1.2; young: *M* = 7.6%, SE = 1.4; *t*_(37)_ = 1.9, *p* = 0.064). In contrast, the Older1 group had significantly smaller congruency effects compared to the pooled young groups: Instructed RT (Older1: *M* = 14.4 ms, SE = 4.0; young: *M* = 44 ms, SE = 3.3; *t*_(35)_ = 5.23, *p* < 0.001), Free-choice RT (Older1: *M* = 1.6 ms, SE = 2.6; young: *M* = 29.9 ms, SE = 4.0; *t*_(35)_ = 4.4, *p* < 0.001) and Instructed error rate (Older1: *M* = 1.9%, SE = 1.4; young: *M* = 7.6%, SE = 1.4; *t*_(35)_ = 2.89, *p* = 0.007). To summarize, we found a smaller congruency effect in Older1 group compared to the younger groups but not in Older2.

Thus, as observed in our earlier study on the Young2 group (Furstenberg et al., 2015), masked primes affected Young1 and Older2 participants’ responses despite the fact that they were not consciously detectable. The prime effect was smaller and bordering significance in the Older1 group in which the prime was presented for 17 ms. Taken together, the behavioral results confirmed our previous finding (Furstenberg et al., 2015) that incongruent subliminal primes interfere with the process of initiating and executing a motor plan, whether triggered by an external cue (in the Instructed condition) or by an internal plan (in the Free-choice condition). In the current study we see that these effects are present in older participants as well.

### EEG

**FIGURE 3:**
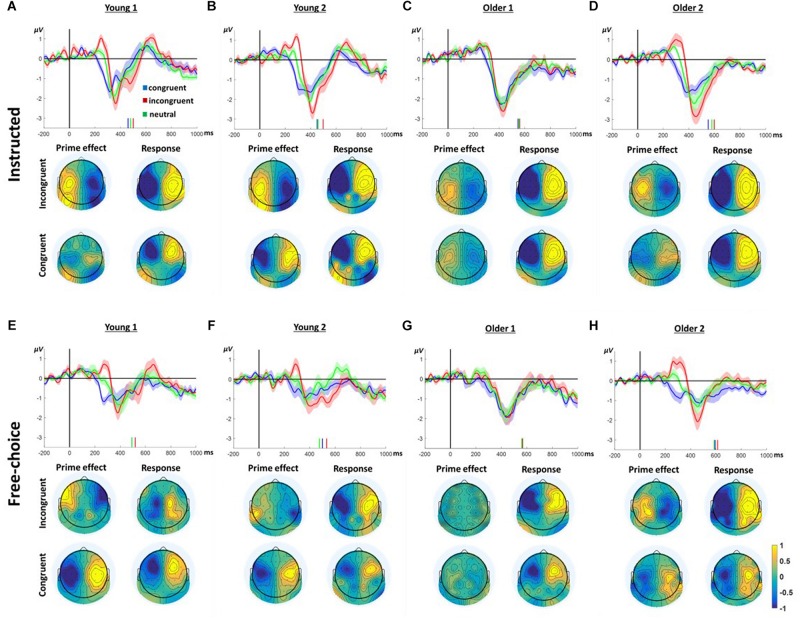
LRP results in Instructed **(A–D)** and Free-Choice trials **(E–H)**. In each panel, the waveform depicts the stimulus-locked group average LRP_across hands_ at channels C3/4 for congruent, incongruent, and neutral prime trials. Zero indicates prime onset. Downward deflection indicates preparation toward the final response direction. Upward deflection indicates preparation in the opposite direction. The shaded area around the mean indicates 95% confidence interval across participants. Short vertical lines are the congruent, neutral, and incongruent mean RTs. The topographies below show the “LRP_across hands_” distribution for all electrodes based on the formula in section “ERP Analysis.” Note that by the nature of the “LRP_across hands_” calculation, the maps are left–right symmetrical up to the sign. Prime effect window was defined around the positive peak of the incongruent response. The Response window was defined around the negative peak prior to the response.

Figure 3 presents the LRP responses in all conditions and groups. Considering the way the LRP is calculated, negative deflection of the LRP reflects greater negativity contralateral to the ultimately responding hand. In contrast, a positive deflection reflects greater negativity ipsilateral to the ultimately moving hand, presumably reflecting preparation to move the other hand. Indeed, prior to the response time (indicated by the vertical bars), all groups and conditions evince a negative deflection peaking ∼100ms before the response. However, prior to this final contralateral negativity, all groups, except the Older1 group, present a ChoI pattern, as defined in our previous study, in the window of ∼200–350 ms following the prime onset. Whereas the congruent prime condition is characterized with negativity during this window, the response to the incongruent prime is characterized by a positive deflection, which then reverses to the direction of the hand that finally moved. This is consistent with a preparation to move the ultimately responding hand following congruent primes, and an initial preparation to move the other hand following incongruent primes. Taken together, this pattern is commensurate with an initial intention to move based on the masked prime. The neutral-condition response is intermediate between the responses to congruent and incongruent primes, consistent with intermediate preparation time following non-informative primes. Since the LRP was calculated based on a weighted average of left and right hand responses, and the Free-choice conditions included unequal number of left and right hand responses, a lateralized response may be due to a permanent asymmetry which does not depend on the hand responding. To ensure that the ChoI pattern is genuinely a true LRP (i.e., hand dependent), we recalculated the LRP based on an unweighted average of the left and right responses, and obtained a similar pattern (Supplementary Figure S2). In the current study we were mainly interested in comparing this effect between different age groups. Specifically, we compared the manner in which, within each group, the congruent and incongruent trials differed in this “prime effect” temporal window.

The difference between congruent and incongruent trials (i.e., the ChoI pattern) is robust in the Young1, Young2 (from our previous study) and Older2 groups in both the Instructed and Free-choice conditions (Figure 3; “prime effect” temporal window cluster-based permutation analysis; Young1: Instructed 227–314 ms, *p* = 0.009, Free-choice 236–323 ms, *p* = 0.002; Young2: Instructed 199–339 ms, *p* = 0.01, Free-choice 265–330 ms, *p* = 0.04; Older2: Instructed 275–382 ms, *p* = 0.01, Free-choice 223–362 ms, *p* < 0.001). In the Older1 group, which was exposed to the shorter prime, the EEG effect was weak, significant only in the Instructed condition (294–357 ms, *p* = 0.027, cluster-based permutation analysis; Figure 3C).

We ran a 2-way ANOVA on the LRP mean amplitudes of 50ms surrounding the center of each group’s “prime effect” significant temporal window, with factors of Congruency (congruent, incongruent, neutral; within-subject) and Group (Young1, Young2, Older1, Older2; between-subjects) separately for the Instructed and Free-choice conditions. The results showed a significant main effect of Congruency (Instructed LRP: *F*(2,92) = 64.3, *p* < 0.001, partial η^2^ = 0.58, Greenhouse-Geisser epsilon = 0.74; Free-choice LRP: *F*(2,92) = 48.1, *p* < 0.001, partial η^2^ = 0.51), with the incongruent LRP amplitude being larger than the congruent LRP amplitude, and the neutral LRP amplitude intermediate. Congruency interacted with Group in the Free-choice condition but not in the Instructed (Instructed LRP: *F*(6,92) = 1.1, *p* = 0.38, partial η^2^ = 0.07; Free-choice LRP: *F*(6,92) = 3.3, *p* = 0.005, partial η^2^ = 0.18). The main Group effect was not significant in either condition (Instructed LRP: *F*(3,46) = 0.9, *p* = 0.43, partial η^2^ = 0.06; Free-choice LRP: *F*(3,46) = 1.4, *p* = 0.25, partial η^2^ = 0.09). Following the significant interaction between Group and Congruency, pairwise contrasts within the Congruency factor (Congruent vs. Incongruent, Congruent vs. Neutral, Neutral vs. Incongruent) for each group revealed that in Older2, Young1 and Young2 groups, all congruency levels differed significantly from each other, in both the Instructed and Free-choice conditions. In contrast, in the Older1 group the difference between Instructed Neutral and Incongruent was not significant, and neither were any of the differences in the Free-choice condition (Supplementary Table S1). This is commensurate with the weak behavioral effects found in group Older1.

Finally, to examine the pattern of laterality across the scalp, we drew topographic maps of the “LRP” for all electrodes; i.e., every electrode was calculated as the difference between its value and its homolog electrode value, following the unweighted LRP formula (section “ERP Analysis”). Figure 3 depicts the topographies of this laterality effect across the scalp. The left and right side of these maps are by definition identical with reversed signs. The critical comparison is between the distribution of laterality during a 40 ms window centered on the peak of the prime effect (ChoI) and the distribution during a 40 ms window centered on the negative peak of the LRP just before the actual button press, both based on the incongruent condition. Overall the topographies show that the pattern of laterality indeed flips – that is, the inter-hemispheric asymmetry of the early deflection in the incongruent case is roughly a mirror image of the topography just before the actual movement, reflecting the ChoI.

### EMG

In a previous study we found evidence for ChoI also in the form of increased probability of hand muscle EMG activity in the non-responding hand (“spurious activation”) in incongruent vs. congruent trials in the Young2 group (Furstenberg et al., 2015). We ran a permutation test for 2-way analysis of variance (10,000 permutations) with factors Congruency (congruent, incongruent; within-subject) and Group (young (pooled), older (pooled); between-subjects) for the probability of a spurious activation, separately for the Instructed and Free-choice conditions. Since the permutation-ANOVA test was implemented in a way that required groups to be equal in size, we had to eliminate from the analysis two young participants in each run of the test (given that *N*_young_ = 26 and *N*_older_ = 24). This was done by running the permutation-ANOVA 325 times (combinations of 2 out of 26), removing in each run two young participants, until all possible couples were removed. Therefore, we obtained 325 assessments of significance, and report below the percentage of times the effects were significant at an alpha level of 0.05 relative to the null distribution. The results showed a significant main effect of Congruency (Instructed: *p* < 0.05 for 100% of the runs; Free-choice: *p* < 0.05 for 100% of the runs), an unstable effect of Group (Instructed: *p* < 0.05 for 65% of the runs; *p*-value range: 0.02 < *p* < 0.15; Free-choice: *p* < 0.05 for 82% of the runs; *p*-value range: 0.015 < *p* < 0.11), and a significant interaction (Instructed: *p* < 0.05 for 100% of the runs; Free-choice: *p* < 0.05 for 95% of the runs).

**FIGURE 4:**
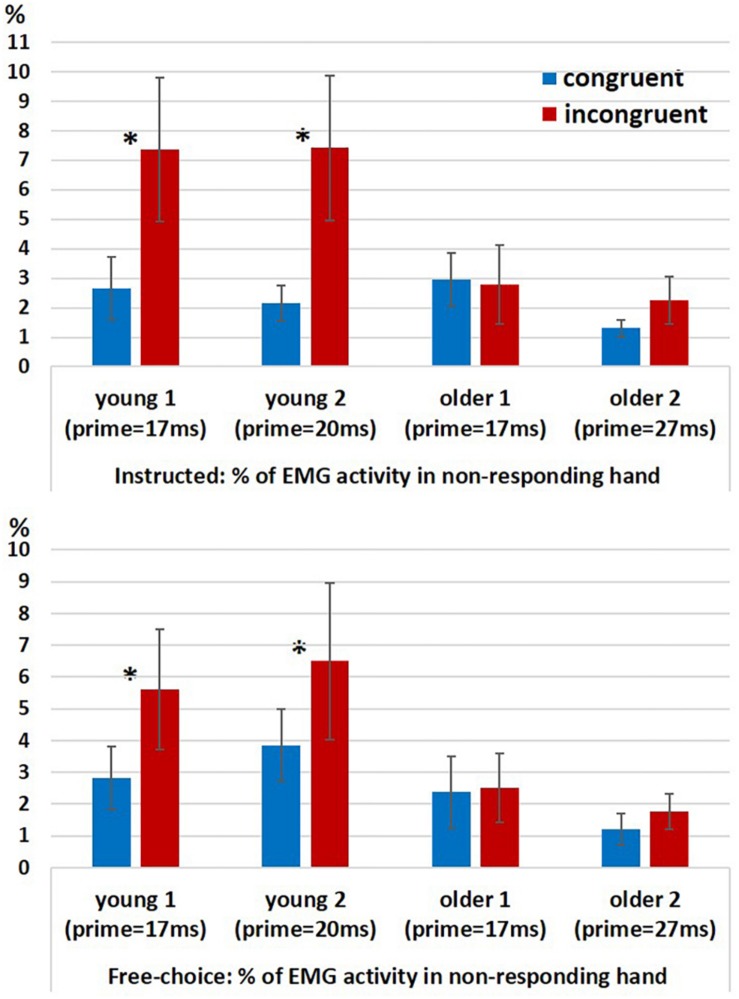
EMG results. Percentage of EMG responses in the non-responding hand in Instructed trials (top panel) and Free-choice trials (bottom panel) in congruent and incongruent conditions within each group (Young1, Young2, Older1, and Older2). Error bars denote the standard error.

In order to understand the source of interaction, we compared congruent and incongruent conditions within each group. The percentage of trials with spurious activation was significantly larger in the incongruent conditions than in the congruent conditions only in the younger groups but not in the older groups, for both Instructed and Free-choice conditions (permutation-based paired test within each group for Instructed and Free-choice conditions; Young1: *p* = 0.002 and *p* = 0.009, respectively; Young2: *p* = 0.002 and *p* = 0.027, respectively; Older1: *p* = 0.43 and *p* = 0.39, respectively; Older2: *p* = 0.55 and *p* = 0.08, respectively; Figure 4).

### Analysis of Instructed Neutral Trials

#### EEG

**FIGURE 5:**
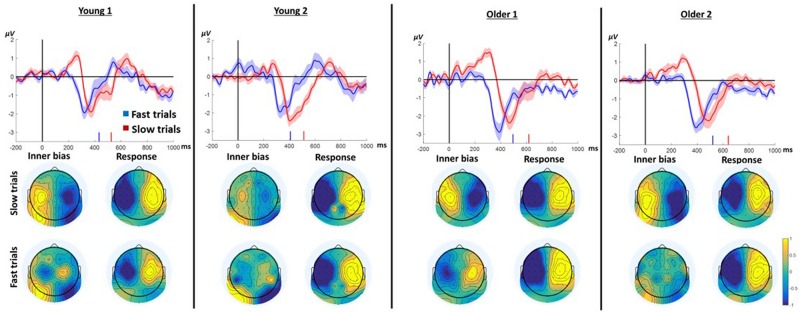
LRP results for Instructed *neutral* trials (NRR, NLL) divided into fast and slow trials (median split) pooling left and right responses. In each panel, the waveform depicts the stimulus-locked group average LRP_across hands_. Zero indicates prime onset. Downward deflection indicates preparation toward the final response direction. Upward deflection indicates preparation in the opposite direction. The shaded area around the mean indicates 95% confidence interval across participants. Short vertical lines on the *x* axis are the fast and slow trials mean RTs. The topographies below show the “LRP_across hands_” distribution for all electrodes based on the formula in section “ERP Analysis.” Note that by the nature of the “LRP_across hands_” calculation, the maps are left–right symmetrical up to the sign. The “Inner bias” window was defined around the positive peak of the slow trials average. The Response window was defined around the negative peak prior to the response.

We hypothesized that slow responses in the neutral prime conditions result from an endogenous trial-specific (i.e., varying between trials) tendency to use one of the arms which conflicts with the eventual cue to move in another direction. We further surmised that if that were the case, comparing slow to fast responses within neutral trials should resemble the comparison between incongruent and congruent trials in the case of informative primes. Confirming this hypothesis, the LRP signature of ChoI was observed in the Instructed neutral condition (NRR, NLL) in the slow response trials compared to fast response trials in all age groups (Figure 5; slow vs. fast cluster-based permutation analysis; Young1: 187–329 ms, *p* < 0.001; Young2: 265–348 ms, *p* = 0.019; Older1: 208–411 ms, *p* < 0.001; Older2: 292–418 ms, *p* = 0.002). A 2-way ANOVA was conducted on the neutral Instructed LRP mean amplitudes of 50ms surrounding the center of each group’s significant temporal window, with factors RT (Fast, Slow; within-subject) and Group (Young1, Young2, Older1, Older2; between-subjects). The results showed a significant main effect of RT [*F*(1,46) = 81.3, *p* < 0.001, partial η^2^ = 0.64], with the Slow LRP amplitude being larger than the Fast LRP amplitude. Nevertheless, there was no interaction between RT and Group [*F*(3,46) = 0.6, *p* = 0.61, partial η^2^ = 0.04]. There was a significant main effect of Group [*F*(3,46) = 6.96, *p* = 0.001, partial η^2^ = 0.31], grouping together Young1 and Older1 on the one hand, and Young2 and Older2 on the other hand. However, we had no hypothesis regarding this result.

To examine the pattern of laterality across the scalp, we drew as before topographic maps of the “LRP” for all electrodes (see section “EEG”). The critical comparison is between the distribution of laterality during the ChoI period (40 ms centered around the positive peak of the Slow trials), and the distribution just before the actual button press (mean of a 40ms window around the peak negativity of the response to Slow and Fast trials, respectively). The topographies show that the pattern of laterality indeed flips – that is, the inter-hemispheric asymmetry of the early deflection in the Slow case is roughly a mirror image of the topography just before the actual movement, reflecting the ChoI (Figure 5).

The hypothesis that the ChoI is caused in this case by a conflict with an endogenous early intention rather than with the prime, explains why even the Older1 group, which did not have a large ChoI signal in the incongruent Instructed and Free-choice conditions, likely because of the short prime duration, elicited a clear ChoI signal in the analysis of neutral primes.

#### EMG

**FIGURE 6:**
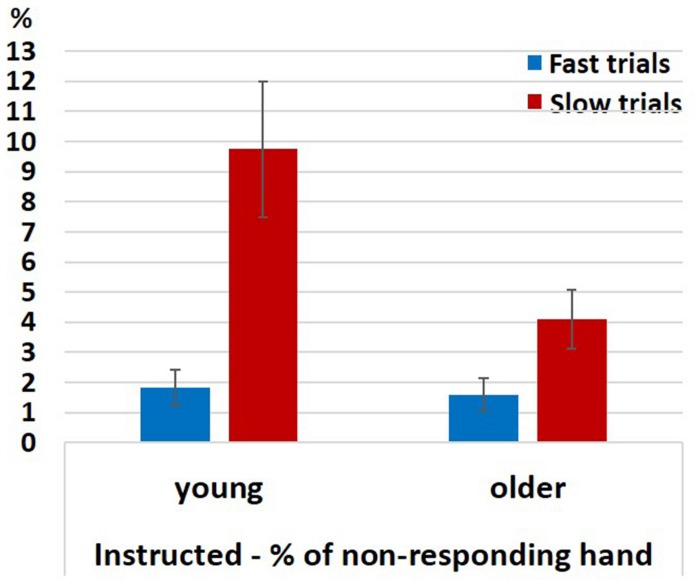
Percentage of EMG responses in the non-responding hand in fast and slow Instructed neutral trials divided according to a median split. Error bars denote the standard error.

For analyzing the EMG signals of the non-responding hand in the neutral Instructed condition we ran a permutation test for 2-way analysis of variance (10,000 permutations) with factors RT (Fast, Slow; within-subject) and Group (young (pooled), older (pooled); between-subjects) for the probability of a spurious activation. As explained in section “EMG” the permutation-ANOVA test was implemented in a way that required the groups to be equal in size, therefore, we ran the test 325 times eliminating from the analysis two young participants in each run of the test (since *N*_young_ = 26 and *N*_older_ = 24). The results showed a significant main effect of RT (*p* < 0.05 for 100% of the runs), an unstable effect of Group (*p* < 0.05 for 27% of the runs; *p*-value range: 0.03 < *p* < 0.22), and a significant interaction (*p* < 0.05 for 100% of the iterations). The effect of RT resulted from more spurious activations in slow than in fast RTs. A permutation-based paired test revealed that this was the case both within the young (pooled together) and within the older (pooled together) participants (Figure 6; permutation-based paired test; young: *p* < 0.001, older: *p* < 0.001). The significant interaction reflects the fact that in the slow trials the probability for spurious activations was larger in the younger participants (pooled together) compared to the older participants (pooled together) (Mann–Whitney *U* test, *z* = 2, *p* = 0.045), but not in the fast trials (Mann–Whitney *U* test, *z* = 0.48, *p* = 0.65).

## Discussion

The present study investigated the effects of aging on the process of intention formation and ChoI using a masked prime paradigm. We hypothesized that an invisible prime will instigate an intention to respond with the respective hand, and used the LRP response derived from EEG, to follow change of this intention following either visible instructions or an endogenous decision. The current results replicate our previous results of ChoI in a picking situation with a larger number of participants spanning the range from young adulthood to late middle-age. Across the board, an implicit initial preparation to act in one way, either induced endogenously or exogenously, could be overcome either by an exogenous cue or by endogenous “free” will. The invisible prime affected multiple behavioral measures within both young and older groups: the RT in the Instructed and Free-choice condition, the error rates in the Instructed condition and the percent of congruent button presses in the Free-choice condition. In all groups an initial intention to move one of the hands was formed, up to the level of motor\premotor cortex, as reflected by the LRP, and in some cases even down to muscle activation (either tacitly, revealed by the EMG of the non-responding hand, or overtly as an erroneous button press). Nevertheless, all groups were able to implement control over this initial intention and reverse it. Such change causes a slowing of RT, and is reflected in LRP reversal. This suggests that in the older group studied here, there is no detectable decline in the capacity to quickly change intentions, even when the internal motivations or external conditions change very rapidly (∼50 ms from prime to cue).

### Change of Intention

Whereas the ChoI is to be expected in the case of a prime followed by an incongruent instructive cue, it is surprising to find it in the Free-choice condition, a picking situation in which on an explicit or reason-based level there is symmetry between the picking alternatives (pressing the right or left button). It has been assumed that the decision process is based in such cases on a lower, implicit level, in which the symmetry is broken by low level perturbations [this is Leibniz’s approach as opposed to Newton’s (Leibniz, 1981/1765); for Newton’s reply to Leibniz see Ariew (2000), fourth reply; Details on this controversy can be found in Furstenberg et al. (2014)]. However, our results indicate that although neuronal asymmetry was exogenously induced by the masked prime, and an intention to use one of the hands was indeed formed, as expressed by the LRP or even EMG, participants in many cases acted against this established asymmetry and formed an opposing intention (ChoI signature). The current results show that this capacity to overcome a formed intention is similar across the age groups. In line with recent studies showing that inhibition is preserved in older individuals in many tasks (see “Introduction” section), our results indicate that the inhibition involved in the ChoI process at the cortex level seems to be the same between the older and younger participants at least in the age groups we studied. Note that the self-inhibition of NCE is not observed in our study in any age group due to the fact that the interval between prime and cue was not long enough.

In both younger and older groups, we observed the ChoI signature not only in cases in which the initial intention was induced by a prime, but also in cases in which it was induced by an endogenous bias. Dividing the neutral Instructed trials (i.e., instructing cue with no directional prime) into fast RT and slow RT trials, revealed that the slow neutral responses are correlated with a ChoI signature in both the younger and the older groups. Our results suggest that, when there is no directional prime, slow responses are caused, at least partially, by an endogenous intention to move in a certain direction (observed by the LRP), which is overcome by a directional cue to move the other hand, resulting in a ChoI signature.

The effects of the prime–cue congruency were much weaker in the Older1 group than in all other groups. This was very likely due to the short prime duration used in this group (17 ms), which was probably below the minimal duration needed for this age group. The fact that indeed it was this perceptual limitation that caused the difference between the two older groups is supported by the analysis of the trials with neutral prime (see paragraph above). The Older1 group expressed the same ChoI signature as the other groups when intention was based on endogenous processes instead of perceptual ones. Note that the short prime duration was the same for the Older1 and Young1 groups, meaning that the older participants needed more time for processing the prime. An important cautionary note from this observation is that we could erroneously conclude that older individuals have less ChoI whereas in fact their limitation is probably perceptual.

### Speed-Accuracy Tradeoff

The comparable dynamics of ChoI in the younger and older groups was found despite the clear difference between the groups in their behavioral regimes. The two groups employed a different speed-accuracy tradeoff: young participants were fast but produced more errors in the incongruent conditions, whereas older individuals were slow but more accurate (cf. Starns and Ratcliff, 2010; Lamb et al., 2016). Observing that healthy older adults were less likely than younger adults to sacrifice accuracy for speed, it was suggested that, at least partially, the older adults’ slowing may be a learned adaptive strategy (Lamb et al., 2016), for example in order not to trip or fall. This prudence is consistent with findings of reduced risk seeking with age [cf. West et al. (2014), in an older group, but see Mata et al. (2011) for more complex findings regarding the relation between risk seeking and age]. It is also possible that the older participants are less confident, and therefore, accumulate more evidence in order to make a decision (cf. Tinetti et al., 1994; Chamberlin et al., 2005; Jung et al., 2015).

### Serial or Continuous Signal Flow?

Many studies on decision-making have suggested a decision criterion with an accumulator threshold crossing model (e.g., Bowman et al., 2006; Bogacz et al., 2007; Mattler and Palmer, 2012; Schurger et al., 2012). As mentioned in the Introduction, this simple model is challenged by the phenomenon of ChoI, since under this model a formed intention to move in a certain direction would mean that the criterion threshold has been crossed, and there is no room for reversal. However, similar to previous findings (e.g., Coles et al., 1985; Smid et al., 1990; Burle et al., 2008; Roger et al., 2014), our results clearly indicate that there is a phenomenon of change of a movement preparation that has flowed downstream into the motor system. Selen et al. (2012) proposed an alternative approach to the accumulator threshold crossing model, consisting of a more continuous process, by which “the decision process may provide a continuous flow of information to the motor system, allowing it to prepare in a graded fashion for the probable outcome.” According to this approach the accumulated evidence for the decision is continuously represented in the human motor system (Kubanek and Kaplan, 2012). These conflicting hypotheses echo an earlier dispute whether the sensory-motor system is best described by a “serial signal flow” model in which the information processing stages are activated one at a time upon the completion of processing of the preceding stage, or by a “continuous signal flow” scenario, in which the output of any processing stage is continuously available to all subsequent, or concurrent, processes along recurrent and feedback loops (Sternberg, 1969; Grice et al., 1977; Eriksen and Schultz, 1979; Coles et al., 1985; Smid et al., 1990). A later version of this dispute appears between those holding a serial view of perception, cognition and action stages, and those holding an “affordance competition hypothesis” (Cisek, 2007; see review in Cisek and Kalaska, 2010).

Our findings, in this study and its predecessor, of EMG “spurious activation” in the non-responding hand as part of a ChoI process, together with previous studies showing such activations (e.g., Coles et al., 1985; Smid et al., 1990; Zeef and Kok, 1993; Burle et al., 2008; Roger et al., 2014), support the continuous signal flow hypothesis (cf. Resulaj et al., 2009; Selen et al., 2012). Notably, our findings extend this notion to the case of subliminal priming (cf. Schmidt, 2002), as well as to “free choice” picking situations where there should be no rational reason to switch from a movement that in fact already started executing at the muscular level to its alternative.

Finally, the behavioral difference between younger and older adults manifested not only in lower error rates in the Instructed condition, but also in fewer “near misses,” evident in reduced occurrences of EMG activations in the non-responding hand. We suggest that the older are “wiser” in that they first reach a final decision and only then send the command to the peripheral motor system, perhaps due to reduced confidence in the ability to withhold action in case of error. In contrast, in the younger groups there is a flow of information downstream during the decision process, thus revealing a ChoI phenomenon also in the peripheral muscle level. Thus, it seems that as people age, there is a move along the continuum between continuous and serial signal flow, which allows optimal interaction with the environment.

## Ethics Statement

This study was carried out in accordance with the recommendations of the Declaration of Helsinki with written informed consent from all subjects. All subjects gave written informed consent in accordance with the Declaration of Helsinki. The protocol was approved by the University of California, Berkeley Committee for Protection of Human Subjects.

## Author Contributions

AF, HS, RTK, and LYD conceived and designed the study and interpreted the results. AF and CDD collected the data. AF analyzed the data and drafted the manuscript. HS, RTK, and LYD provided critical revisions. All authors approved the submitted version of the manuscript.

## Conflict of Interest Statement

The authors declare that the research was conducted in the absence of any commercial or financial relationships that could be construed as a potential conflict of interest.

## Footnotes

1 We use the term “intention” as a plan to execute a course of action. This should be distinguished from such things as (more general) urges, desires and wishes. “Proximal intention” is defined as an immediate intention to do something now, in contrast to long-term commitments and intentions (Mele, 2009; Furstenberg, 2014).

2 http://www.biosemi.com/pics/cap_64_layout_medium.jpg

3 In our previous study, the neutral trials in the free choice condition had shorter RTs than the congruent trials. Hence our directional a-priori hypothesis is not supported here.
