# Supplementary material for: Effect of Aging on Change of Intention
Source: Front Hum Neurosci. 2019 Jul 31;13:264. doi: 10.3389/fnhum.2019.00264 (PMC6685419; doi:10.3389/fnhum.2019.00264)
Supplement: Supplementary file 1 [file Table_1.docx]

**Table S1.** P-values in pairwise comparisons of the LRP amplitude in the Congruency factor within each group.

|  | **Instructed** |  |  |  |  |  |  |  |  |
| --- | --- | --- | --- | --- | --- | --- | --- | --- | --- |
|  | **Young1** |  | **Young2** |  | **Older1** |  | **Older2** |  |  |
|  | **incongruent** | **neutral** | **incongruent** | **neutral** | **incongruent** | **neutral** | **incongruent** | **neutral** |  |
| **congruent** | 0.000 | 0.000 | 0.000 | 0.000 | 0.01 | 0.02 | 0.000 | 0.000 |  |
| **incongruent** |  | 0.003 |  | 0.004 |  | 0.11 |  | 0.000 |  |
|  |  |  |  |  |  |  |  |  |  |
|  | **Free-choice** |  |  |  |  |  |  |  |  |
|  | **Young1** |  | **Young2** |  | **Older1** |  | **Older2** |  |  |
|  | **incongruent** | **neutral** | **incongruent** | **neutral** | **incongruent** | **neutral** | **incongruent** | **neutral** |  |
| **congruent** | 0.000 | 0.000 | 0.000 | 0.019 | 0.28 | 0.59 | 0.000 | 0.000 |  |
| **incongruent** |  | 0.005 |  | 0.007 |  | 0.49 |  | 0.01 |  |
|  |  |  |  |  |  |  |  |  |  |
