# Supplementary material for: Effect of Aging on Change of Intention
Source: Front Hum Neurosci. 2019 Jul 31;13:264. doi: 10.3389/fnhum.2019.00264 (PMC6685419; doi:10.3389/fnhum.2019.00264)
Supplement: Supplementary file 2 [file Table_2.DOCX]

**Supplementary figure legend**

**Supplementary figure S1**. Free-choice RT distribution.

**Supplementary figure S2**. Comparison of weighted and unweighted LRPs. The LRP_across hands_ was calculated based on a weighted average of left and right hand responses. To ensure that the ChoI pattern is genuinely a true LRP (i.e. hand dependent), we recalculated the LRP_across hands_ based on an unweighted average of the left and right responses. If the lateralized response were a hand-independent fixed bias, it should be absent in the unweighted average. Note that in the free choice condition the number of left hand and right hand responses was not necessarily equal, and thus the unweighted response is noisier than the weighted response (which considers the number of trials). However, the lateralized responses remain unaltered in the two types of LRP calculation.
