# Supplementary figures and images for: Effect of Aging on Change of Intention
Source: Front Hum Neurosci. 2019 Jul 31;13:264. doi: 10.3389/fnhum.2019.00264 (PMC6685419; doi:10.3389/fnhum.2019.00264)

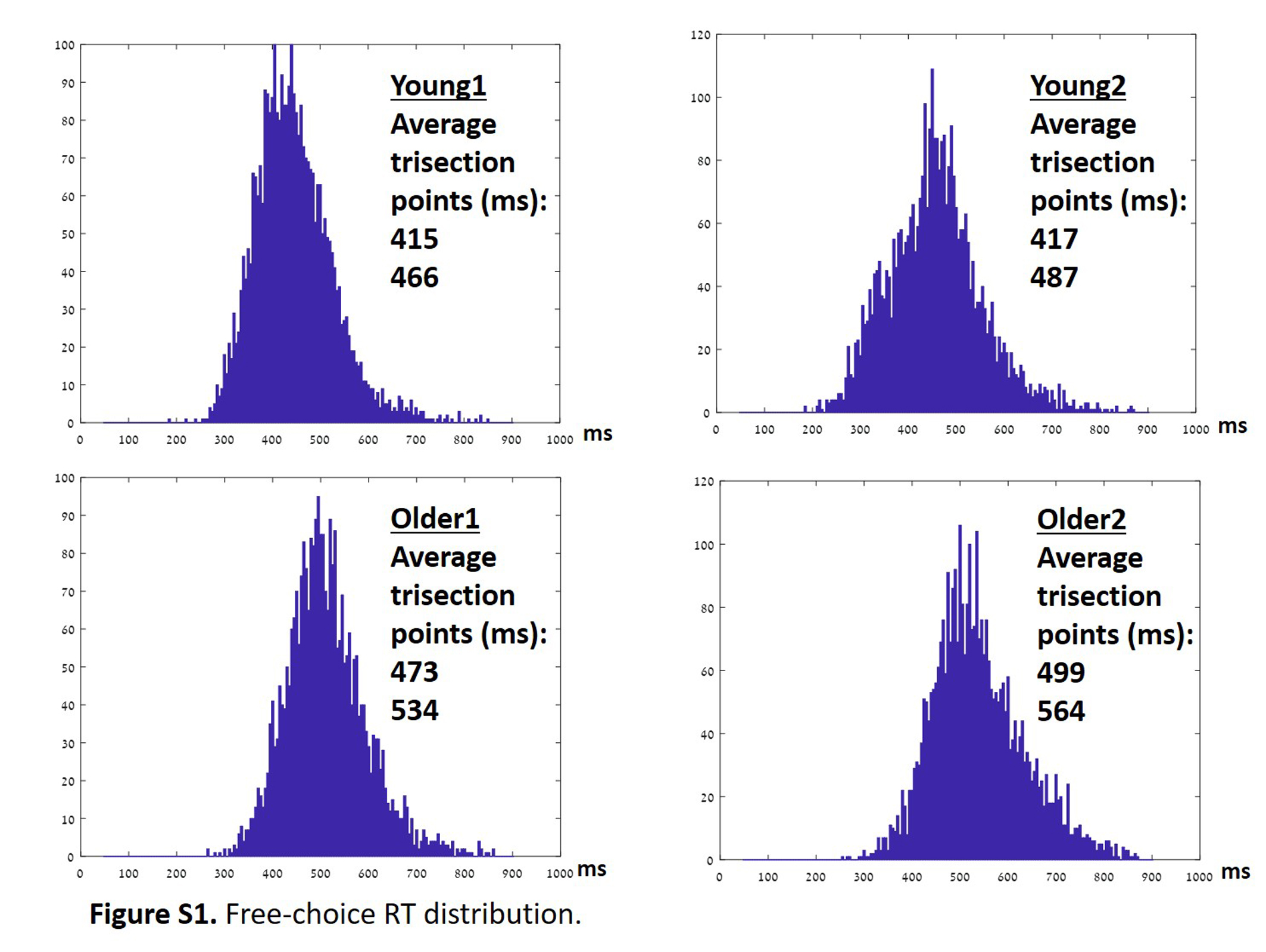

Supplement: Supplementary file 3 [file Image_1.jpeg]

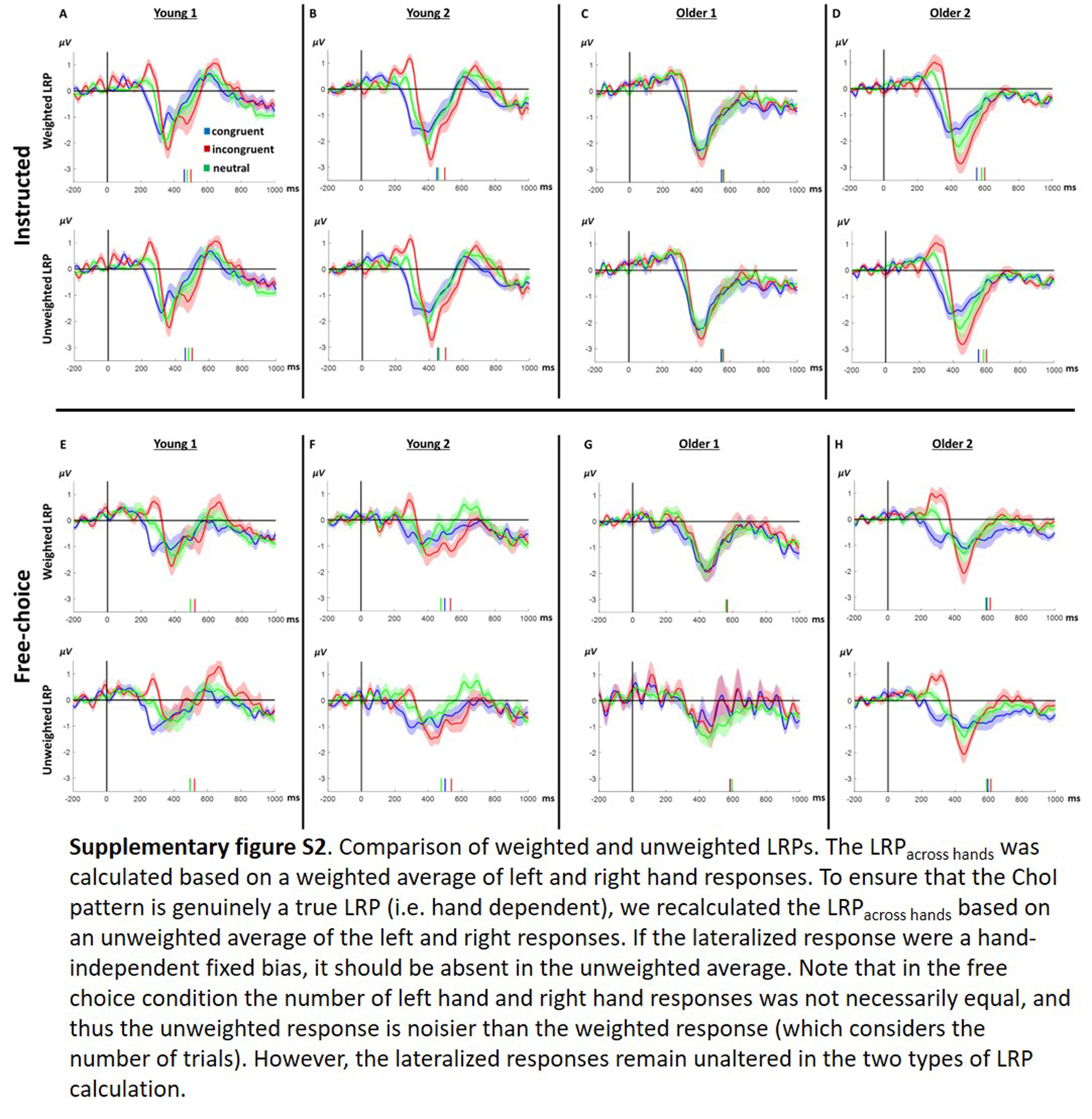

Supplement: Supplementary file 4 [file Image_2.jpg]
